# The impact of preterm birth <37 weeks on parents and families: a cross-sectional study in the 2 years after discharge from the neonatal intensive care unit

**DOI:** 10.1186/s12955-017-0602-3

**Published:** 2017-02-16

**Authors:** Ashwini Lakshmanan, Meghana Agni, Tracy Lieu, Eric Fleegler, Michele Kipke, Philippe S. Friedlich, Marie C. McCormick, Mandy B. Belfort

**Affiliations:** 1 Center for Fetal and Neonatal Medicine, USC Division of Neonatal Medicine, Children’s Hospital Los Angeles; Keck School of Medicine, University of Southern California, 4650 Sunset Boulevard, MS #31, CA 90027 Los Angeles, USA; 2 Newborn and Infant Critical Care Unit, Children’s Hospital Los Angeles, 4650 Sunset Boulevard, MS #31, CA 90027 Los Angeles, USA; 3 Department of Preventive Medicine, Keck School of Medicine, University of Southern California, Los Angeles, CA USA; 4 Center for Health Policy and Economics, University of Southern California, Los Angeles, CA USA; 5 Drexel School of Medicine, Philadelphia, PA USA; 6 Division of Research, Kaiser Permanente, Oakland, CA USA; 7 Division of Emergency Medicine, Boston Children’s Hospital, Boston, MA USA; 8 Saban Research Institute, Children’s Hospital Los Angeles, Los Angeles, CA USA; 9 Division of Newborn Medicine, Boston Children’s Hospital, Boston, MA USA; 10 Beth Israel Deaconess Medical Center, Boston, MA USA; 11 Department of Social and Behavioral Sciences, The Harvard T.H. Chan School of Public Health, Boston, MA USA; 12 Department of Pediatric Newborn Medicine, Brigham and Women’s Hospital, Boston, MA USA

**Keywords:** Impact on family, Impact on parents, Prematurity, High-risk infant, Post-discharge

## Abstract

**Background:**

Little is known about the quality of life of parents and families of preterm infants after discharge from the neonatal intensive care unit (NICU). Our aims were (1) to describe the impact of preterm birth on parents and families and (2) and to identify potentially *modifiable* determinants of parent and family impact.

**Methods:**

We surveyed 196 parents of preterm infants <24 months corrected age in 3 specialty clinics (82% response rate). Primary outcomes were: (1) the Impact on Family Scale total score; and (2) the Infant Toddler Quality of Life parent emotion and (3) time limitations scores. Potentially modifiable factors were use of community-based services, financial burdens, and health-related social problems. We estimated associations of potentially modifiable factors with outcomes, adjusting for socio-demographic and infant characteristics using linear regression.

**Results:**

Median (inter-quartile range) infant gestational age was 28 (26–31) weeks. Higher Impact on Family scores (indicating worse effects on family functioning) were associated with taking ≥3 unpaid hours/week off from work, increased debt, financial worry, unsafe home environment and social isolation. Lower parent emotion scores (indicating *greater* impact on the parent) were also associated with social isolation and unpaid time off from work. Lower parent time limitations scores were associated with social isolation, unpaid time off from work, financial worry, and an unsafe home environment. In contrast, *higher* parent time limitations scores (indicating *less* impact) were associated with enrollment in early intervention and Medicaid.

**Conclusions:**

Interventions to reduce social isolation, lessen financial burden, improve home safety, and increase enrollment in early intervention and Medicaid all have the potential to lessen the impact of preterm birth on parents and families.

**Electronic supplementary material:**

The online version of this article (doi:10.1186/s12955-017-0602-3) contains supplementary material, which is available to authorized users.

## Background

In the United States, nearly 500,000 infants, or 11.7% of all live births, are born preterm (<37 weeks’ gestation) each year [1, 2]. Preterm birth and the sometimes associated prolonged newborn hospitalization are great family stressors, and can lead to subsequent family dysfunction [3–5].

All preterm infants are at risk for re-hospitalization, as well as medical and neurodevelopmental complications, even moderate to late preterm infants (born at 32 to <37 weeks’ gestation) [6]. A particularly challenged sub-group is very low birth weight (VLBW) infants or those born < 1500 g. More than 90% of VLBW infants are discharged home from the neonatal intensive care unit (NICU). The burden of continued health and developmental problems faced by these infants is substantial [7–9]. For example, compared with normal birth weight children, VLBW children face a 2–3 fold greater risk for visual and hearing impairment, speech delays and attention disorders [10, 11]; may have poor feeding and growth, respiratory complications, and face neurocognitive difficulties [12–16].

Given these ongoing problems and risks, families of preterm children often must manage numerous medical and developmental needs above and beyond what is required for a healthy full term infant, for months or even years after the neonatal discharge. For example, during the first year of life, VLBW infants are prone to re-hospitalization and require increased outpatient care [17–19]. Parents must transport their child for medical appointments and therapies, communicate with the child’s pediatrician and other healthcare providers, and are often responsible for daily tasks, such as administering medications and monitoring chronic conditions.

The intensity of care and high level of vigilance required by families to meet the needs of their preterm child makes it likely that having a preterm child adversely affects the quality of life of the parents and the family overall. The 2006 Institute of Medicine’s (IOM) report on Preterm Birth: Causes, Consequences and Prevention stressed the importance of assessing aspects of family and parent quality of life and stress beyond maternal psychological well-being [20–22]. A better understanding of the impact of preterm birth on parent and family quality of life, as well as modifiable factors that predispose parents and families to greater or lesser impact would inform community-based and other structured assistance programs designed to lessen the impact.

**Fig. 1:**
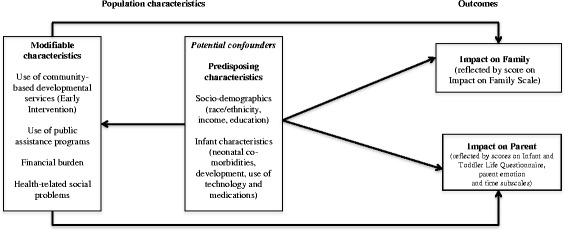
Conceptual Model. Adapted from Phillips [74]

Our main research question was, “Are modifiable characteristics (such as the use of community based and public assistance programs, financial burden, and health related social problems) associated with the impact of preterm birth on parents and families after NICU discharge?” The Anderson and Aday health utilization model [23, 24] provides a useful framework for addressing our research question because it uniquely captures the constructs of access, need, and quality of life. As presented in Fig. 1, we conceptualized potentially modifiable characteristics that influence parent and family impact as: (1) use of community based developmental services and public assistance programs; (2) financial burden; (3) health related social problems. We also specified predisposing characteristics (including socio-demographics and infant health characteristics) related both to modifiable characteristics and to outcomes that could act as confounders.

Our specific aims were (1) to describe the impact of preterm birth on parents and families and (2) and to identify potentially *modifiable* determinants of parent and family impact. Specific variables of interest based on previous literature, were use of community-based resources, financial burden and health-related social problems [25].

## Methods

### Study design and participants

This was a cross-sectional study. We enrolled one parent (mother or father) of preterm (<37 weeks gestation) infants attending 3 outpatient clinics at a large tertiary children’s hospital. One clinic provides multidisciplinary medical and neurodevelopmental follow-up for infants with gestational age <32 completed weeks or birth weight <1500 g discharged from one of 3 large, academic NICU’s and affiliated community-based Level II nurseries, and for more mature or heavier preterm infants with severe medical conditions and/or social risk factors (101 participants enrolled). The second clinic provides pediatric pulmonary care for infants with lung disease that originates in the newborn period, predominantly bronchopulmonary dysplasia (57 participants enrolled). The third clinic provides follow-up care for infants who have suffered neurologic injury during the fetal or newborn period (38 participants enrolled). While some patients were seen at more than one clinic, they were only enrolled once in the study.

We included parents of preterm infants who were up to 24 months corrected age (age from term equivalent). Parents must have been able to answer questions in English or Spanish. If the infant was a multiple, only one response was collected from the family. Study staff provided eligible families with a letter describing the study. Consent was obtained when the parent agreed to complete the questionnaire, which was administered on a laptop (with privacy screens) in the clinic waiting room or examination room. Participants were provided a small incentive to complete the survey.

The Boston Children's Hospital and Children’s Hospital Los Angeles human subjects committees approved the study protocol. Approximately 75% of preterm infants are referred to high-risk infant follow up programs. [3] In this study, of the 239 eligible participants from October, 2011 to June, 2012, 196 completed the questionnaire (82% response rate). The questionnaire is available as Additional file 1.

### Measurements

Measurements of primary outcomes, modifiable characteristics and potential confounders (predisposing characteristics) are summarized in Additional file 2: Table S1.

#### Impact on family

The Impact on Family (IOF) [26–28] measures the global impact of pediatric disability on the family and has been validated on samples of children with chronic health conditions, including preterm birth [25]. The IOF total score is derived from a 27-item questionnaire. For each item, parents indicate the extent to which they agree with a statement regarding the negative impact of the child on the family. Anchors for a 4-point Likert scale were: strongly agree; agree; disagree; and strongly disagree. Examples of IOF items are: “The illness is causing financial problems for the family” and “Our family gives up things because of illness.” IOF subscales include financial impact (8 points), disruption of planning (20 points), caretaker burden (12 points), and familial burden (16 points) for total possible score of 56 points. The total negative impact score served as our summary measure of family burden (**higher** scores indicate **greater** family burden).

In a previous study, internal consistency was high for the overall IOF Scale (Cronbach alphas for total impact, 0.83 to 0.89), but lower for financial (0.68 to 0.79) and coping (0.46 to .52) items [26]. High total scores on the IOF are associated with maternal psychiatric symptoms, poor child health, poor child adjustment, increased child hospitalizations, lower maternal education, and maternal receipt of public assistance [27–29], providing evidence for construct validity.

#### Impact on parent

The Infant Toddler Quality of Life Questionnaire™ (ITQOL) was developed in 1994 for use in children from 2 months to 5 years of age as a “profile measure” for health status and health-related quality of life. ITQOL adopts as its conceptual framework the World Health Organization's definition of health as a state of complete physical, mental and social well-being and not just the absence of disease [30]. It has been used both in randomized clinical trials [31] and observational studies, and is accepted favorably for its ease of use and understandability [32].

In this study, we used the Family Burden scales of the ITQOL, which cover two parent-focused concept subscales, impact-emotion and impact-time, due to caring for their infant or toddler [30, 32, 33]. The parent impact-emotion domain consists of seven items in which the parent is asked to rate how much anxiety or worry each of the child characteristics described in the items has caused during the past 4 weeks (i.e., feeding/sleeping/eating habits; physical health, emotional well being, learning abilities, ability to interact with others; behavior and temperament). The parent impact-time domain consists of seven items in which the parents is asked to rate how much of his/her time was limited for personal needs because of the problems with the child’s personal needs during the past 4 weeks. Internal consistency for the ITQOL parent-impact emotion and parent-impact time scales has been reported in three different populations, a Dutch general population sample (0.61, 0.64) [34], a functional abdominal pain sample (0.72, 0.73) [35] and a burn injury sample (0.79, 0.84) [30, 36].

Raw subscale scores are converted to standardized scores on a 0–100 continuum [37–40]. For each scale, **higher** scores indicate **less** emotional impact and fewer time limitations on the parent (in other words, higher scores represent more favorable outcomes).

#### Potentially modifiable characteristics

##### Use of community-based resources

Participants were asked yes/no questions about the use of community-based developmental resources (such as early intervention programs), use of social services such as food assistance programs, Supplemental Nutrition Assistance Program and the Women, Infant, Children’s program as well as energy assistance/disability programs such the Low Income Home Energy Assistance Program, Transitional Aid to Families with Dependent Children, and receipt of Supplemental Security Income (SSI).

##### Financial Burden

In addition to questions about employment for the participating parent and his/her partner, we asked 6 yes/no questions from the 2007 Commonwealth Fund Biennial Health Insurance Survey [41–44] regarding unexpected costs, increased bills, increased out-of-pocket expenses and financial worry.

##### Health-related social problems

HelpSteps.com is a survey designed to identify health-related social problems. Development of HelpSteps involved literature review and key informant interviews with health and social services experts, yielding an initial list of 25 social domains. Of those, the 5 most relevant domains were identified using a modified Delphi technique: (1) access to health care, (2) housing, (3) food security (4) income security and (5) intimate partner violence [45–48]. Most questions about these domains were adapted from previous surveys (e.g. National Health Interview Survey [49], the American Housing Survey [50], the Philadelphia Survey of Work and Family [51] and the Childhood Community Hunger Identification Project [52]), while a few newly written items were also incorporated into the final HelpSteps survey. In terms of content validity, the domains covered in HealthSteps are well-recognized as being closely tied to health outcomes and costs [53]. HelpSteps is highly effective in identifying problems that can be addressed by referral to appropriate social services [46, 47] and a qualitative study revealed that over 2/3 of participants found the HealthSteps questions to be highly relevant to their own problems [48].

#### Predisposing characteristics (potential confounders)

##### Infant health and development

We obtained information from the medical record regarding delivery and complications during the neonatal hospitalization. We asked parents questions about their infant’s health status since discharge including the number of emergency department visits, monthly clinic appointments, and hospitalizations, immunizations, dependence on technology, and administration of prescription medications.

To assess infant development, we used the Motor and Social Development (MSD) scale [54], which was developed by the National Center for Health Statistics to measure motor, social and cognitive development of young children. Of 48 items derived from standard measures of child development, including the Bayley Scales, Gesell Scale, and Denver Developmental Screening Test, parents complete 15 age-specific items, which ask about specific developmental milestones such as laughing out loud, pulling to stand, and saying recognizable words [55]. We selected the MSD because it is brief and allows for scoring based on a large, national sample [56] with a normative mean of 100 and standard deviation 15, similar to other developmental tests. Higher scores indicate better development. In a previous study of former preterm infants, we showed that the MSD has good internal consistency (Cronbach alpha 0.65-0.88) and is modestly correlated with Bayley Scales of Infant and Toddler Devleopment, 3^rd^ edition, a gold standard professionally administered neurodevelopmental test [56]. Another study reported that infants with lower gestational age at birth have lower scores on the MSD [54]. Although the MSD includes a cognitive, motor, and social subscales, the degree to which it is sensitive to language/communication delays is unknown, which is a potential limitation.

##### Statistical analysis

Our main outcomes were: (1) impact on family total score; (2) impact on parent score determined by the concept of emotion; and (3) impact on parent score determined by the concept of limitation of time. Potentially modifiable determinants included the use of community-based resources, financial burden, and health-related social problems. Potential confounders (predisposing characteristics) were socio-demographics and infant pre-disposing and post-discharge characteristics.

In bivariate analyses, we compared outcome scores across categories of predisposing characteristics (potential confounders) and potentially modifiable determinants. We calculated *p*-values using non-parametric tests (Wilcoxon Rank Sum or Kruskal Wallis). To identify potentially modifiable determinants independent of confounders on our primary outcomes, we created parsimonious multivariable models, adjusting for variables of a priori interest and for other characteristics found to be significant at *p* <0.1. We also examined each model using variance inflation factors (VIF) and did not detect significant collinearity (VIF ≤ 2 for all models).

We used SAS version 9.4 (SAS Institute Inc., Cary, NC) for analyses.

## Results

### Participant characteristics and outcomes

Predisposing characteristics of study participants and outcome measures are shown in Table 1. A majority of participants were white, non-Hispanic (67%). 52% reported an annual household income of ≥ $80,000 and 68% of mothers had attended at least some college. The median (IQR) gestational age of infants at birth was 28 weeks (26–31). The median (interquartile range, IQR) chronologic age of infants at the time of study participation was 10.4 months (7.5-17.2).

**Table 1:** Pre-disposing characteristics and unadjusted associations with impact on family and impact on parent scores (*n* = 196)^a^

		Impact on family^b^		Impact on parent^c^			
		Total score		Emotion score		Time limitations score	
	% of sample	Median (IQR)	*p*-value	Median (IQR)	*p*-value	Median (IQR)	*p*-value
Total cohort		23.5 (18–32)		82 (61–93)		90 (71–100)	
Characteristic							
Time from discharge (months)							
< 6	31	27 (21–35.5)	<0.04	78 (57–93)	<0.04	76 (67–95)	<0.04
≥ 6 to < 12	30	20.5 (17–30)		86 (64–96)		90 (81–100)	
≥ 12 to < 18	24	22 (18–31)		86 (61–93)		90 (71–100)	
≥ 18	15	23 (16–31)		82 (64–96)		90 (67–100)	
Socio-demographics							
*Person completing survey*							
Mother	77	23 (17–30)	0.07	85 (68–96)	<0.01	90 (74–100)	<0.01
Father	23	27.5 (20–36)		69.5 (53–86)		86 (52–90)	
*Race/ethnicity*							
White non-Hispanic	67	24.5 (18–33)	0.2	82 (61–93)	0.6	90 (71–100)	0.1
Hispanic	7	20 (17–23.5)		89 (85.5-93)		100 (88–100)	
Black non-Hispanic	11	26 (18–41)		81 (61–100)		78 (57–100)	
Other	15	22 (17–36)		88 (71–98)		87 (70–94)	
*Income ($/Year)*							
< 40,000	24	24 (19–38)	0.6	86 (69–93)	0.4	90 (71–100)	0.4
≥ 40,000 to <80,000	15	23.5 (17.5-35)		78 (66–96)		90 (52–100)	
≥ 80,000	52	23 (17–30)		82 (61–93)		90 (76–95)	
Missing	9						
*Highest level of education (either parent)*							
≤ High school	32	26 (19–39)	0.2	83.5 (61–93)	0.9	90 (62–100)	0.4
At least some college	68	23 (18–30)		82 (64–93)		90 (76–100)	
*Language*							
Non-English	6	26.5 (20–34)	0.5	85.5 (57–89)	0.9	85 (57–100)	0.8
English	94	24 (18–32)		82 (64–93)		90 (71–100)	
Infant characteristics							
*Birthweight (grams)*							
< 500 to < 1000	53	25 (18–33)	0.06	75 (61–82)	0.2	100 (95–100)	0.2
≥ 1000 to < 1500	24	24 (17–30)		86 (69–96)		84 (62–100)	
≥ 1500 to < 2500	17	18.5 (15–23.5)		86 (76–93)		90 (86–100)	
≥ 2500	6	27 (20–36)		61 (18–90)		77 (33–100)	
*Gestational age (weeks)*							
*<* 24 to < 28	41	26 (18–33)	0.07	82 (66–93)	0.2	88 (67–100)	0.8
≥ 28 to < 32	37	21 (17–28)		85 (66–96)		90 (71–100)	
≥ 32 to < 34	9	26 (18–30)		81 (49–91)		81 (69–95)	
≥ 34 to < 37	13	29 (20–41)		71 (35–93)		81 (57–100)	
*Multiple Birth*							
Yes	15	25 (17–37)	0.44	77 (34–91)	0.7	81 (70–96)	0.7
No	85	23 (18–33)		81 (60–92)		89 (70–99)	
*Neonatal co-morbidities* ^d^							
Yes	59	24 (18–33)	0.5	82 (64–95)	0.6	90 (71–100)	0.7
No	41	23 (17.5-30)		82 (61–93)		90 (67–100)	
*Motor Social Development Score*							
< 85	27	29 (20–36)	0.02	66 (48–91)	0.004	88 (59–98)	0.2
≥ 85	73	23 (17–30)		85 (69–93)		90 (71–100)	
*≥2 clinic appointments/month*							
Yes	74	26 (18–35)	0.0002	79 (57–93)	0.002	90 (67–100)	0.07
No	26	19 (17–24)		89 (75–96)		90 (81–100)	
*At least 1 re-admission to the hospital after discharge*							
Yes	36	31 (20–41)	<0.001	71 (53–89)	0.002	81 (57–95)	0.004
No	64	21 (17–27)		86 (69–96)		90 (76–100)	
*At least 1 visit to the emergency department after discharge*							
Yes	35	26 (18.5-35)	0.1	75 (61–86)	0.004	79 (61.5-96)	0.002
No	65	23 (17–30)		86 (64–96)		90 (76–100)	
*Use of medical technology* ^e^							
Yes	14	40 (29–46)	<0.01	69.5 (53–82)	<0.01	73.5 (52–90)	<0.01
No	86	22 (17–30)		85.5 (66–95)		90 (74–100)	
*Receives at least 1 prescription medication daily*							
Yes	65	27 (20–35)	<0.0001	75 (57–89)	<0.0001	86 (67–95)	0.003
No	35	19 (17–23)		93 (78–100)		94 (81–100)	

^a^ *P*-values derived using Wilcoxon Rank Sum (for 2 sample comparisons) or Kruskal Wallis (for 3 sample comparisons) or Kruskal Wallis H (>3 sample comparison) tests

^b^Derived from Impact on Family Scale (Stein, et al. [27]). Higher score indicates *higher* impact. Impact on Family Scale (range of possible scores, 0 to 56)

^c^Derived from Infant Toddler Quality of Life (ITQOL) questionnaire (Landgraf et al. [30]). Higher score indicates *lower* impact. ITQOL scale (range of possible scores, 0 to 100)

^d^Neonatal co-morbidities include a diagnosis of: fetal growth restriction, surfactant deficiency, necrotizing enterocolitis, intraventricular hemorrhage grade 3 or 4, patent ductus arteriosus, retinopathy of prematurity

^e^Medical technology includes use of: home oxygen, tracheostomy, gastrostomy tube, adaptive wheelchair/stroller

### Unadjusted associations of predisposing characteristics (potential confounders) with outcomes

As shown in Table 1, among pre-disposing characteristics, the use of medical technology, receipt of at least one prescription medication daily, one or more readmission or emergency department visit after neonatal discharge, and 2 or more clinic appointments per month were all associated with greater impact on family, parents, or both. Additionally, having an infant with a low developmental score (MSD < 85) was associated with greater impact on the family and parent emotion. Fathers who completed the survey had higher impact scores than mothers on the parent-focused domains of emotion and time limitations. Of note when we performed an additional sensitivity analysis by running our multivariate models for mothers only, we found our multivariate model estimates were similar in magnitude and direction as the full models that included fathers.

### Potentially modifiable characteristics and unadjusted associations with outcomes

Table 2 shows that use of public housing and public assistance program were associated with greater impact on family. Compensation for time taken off from work was associated with a lower parent emotional score (**less** parental impact) while use of social services, public housing, enrollment in Medicaid and an unsafe home environment were associated with a higher IOF score (**greater** family impact). Markers of financial burden (including unpaid time off work, increased out-of-pocket expenses, bills, debt and financial worry) and social isolation were associated with both greater family and parental impact.

**Table 2:** Potentially modifiable characteristics and unadjusted associations with impact on family and impact on parent scores (*n* = 196)^a^

		Impact on family^b^		Impact on parent^c^			
		Total score		Emotion score		Time limitations score	
Characteristics	% of sample	Median (IQR)	p-value	Median (IQR)	p-value	Median (IQR)	p-value
Use of community-based services							
*Use of a community-based developmental program (early intervention)*							
Yes	92	23 (18–31)	0.9	82 (61–93)	0.9	90 (71–100)	0.2
No	8	28 (17–36)		82 (64–93)		83 (33–95)	
*Use of social services* ^d^							
Yes	45	26 (19–38)	0.02	82 (61–93)	0.9	90 (67–100)	0.8
No	55	23 (17–30)		82 (64–93)		90 (71–100)	
*Use of public housing*							
Yes	9	29 (22–41)	0.04	85 (57–93)	0.7	74 (47–100)	0.4
No	91	23 (18–31)		82 (64–93)		90 (71–100)	
*Receive supplemental security income (SSI)*							
Yes	23	23 (17–31)	0.8	86 (69–100)	0.1	95 (71–100)	0.1
No	77	24 (18–32)		82 (61–93)		90 (71–95)	
*Use a community based clinic for primary care*							
Yes	12	23 (19–41)	0.5	73 (57–96)	0.7	81 (67–100)	0.4
No	88	23 (18–31)		83 (63–93)		90 (71–100)	
Financial Burden since infant discharge							
*Participant employed*							
Yes	68	23 (18–31)	0.7	82 (64–93)	0.9	90 (71–100)	0.4
No	32	24 (18–35)		85 (61–93)		90 (67–100)	
*Partner employed*							
Yes	88	21 (17–30)	0.2	85 (64–96)	0.5	90 (71–100)	0.5
No	12	27 (22–28)		83.5 (64–89)		90 (67–95)	
*Any member has taken ≥ 3 h taken off from work without pay weekly*							
Yes	51	27 (18–40)	<0.005	71 (53–89)	<0.005	81 (52–95)	<0.005
No	49	21 (17–29)		86 (71–96)		90 (80–100)	
*Receives employer-based compensation for time off*							
Yes	31	22 (18–31)	0.6	86 (75–96)	0.02	90 (76–100)	0.2
No	69	24 (17–33)		79 (57–93)		90 (69–100)	
*No compensation (from any source) for time off*							
No compensation	17	29 (18–41)	0.1	65 (50–89)	0.01	85 (47–100)	0.2
Compensation	83	23 (17–30)		85 (68–89)		90 (76–100)	
*Unexpected costs incurred*							
Yes	41	29 (19–39)	<0.01	75 (57–93)	<0.01	81 (59–95)	<0.01
No	59	21 (17–29)		86 (69–96)		90 (76–100)	
*Increased bills*							
Yes	19	30 (23–44)	<0.02	71 (53–86)	<0.02	76 (47–100)	<0.02
No	81	22 (17–30)		86 (68–95)		90 (76–100)	
*Increased out-of-pocket expenses*							
Yes	13	33 (23–44)	<0.04	75 (61–93)	<0.04	76 (43–100)	<0.04
No	87	23 (18–30)		82 (64–93)		90 (71–100)	
*Increased financial worry*							
Yes	59	27 (20–35)	<0.0001	75 (57–93)	<0.0001	81 (62–95)	<0.0001
No	41	20 (17–27)		89 (75–96)		95 (86–100)	
*Collections were discussed prior to discharge*							
Yes	19	30 (21–42)	<0.001	82 (69–93)	0.8	90 (71–100)	0.5
No	81	23 (17–30)		85 (61–93)		90 (71–100)	
*Enrollment in Medi-caid*							
Yes	33	27 (20–38)	0.04	82 (61–93)	0.5	90 (71–98)	0.9
No	67	23 (17–31)		82 (64–93)		90 (71–100)	
Health-related social problems							
*Home safety* ^e^							
Safe	91	23 (17–31)	0.03	85 (64–93)	0.2	90 (71–100)	0.05
Unsafe	9	30 (23–43)		75 (57–89)		76 (43–90)	
*Domestic Violence*							
Reported	1	23	0.9	82	0.9	100	0.2
Not reported	99	24 (18–31)		84 (61–93)		90 (71–100)	
*Social isolation* ^f^							
Yes	16	30 (23–46)	<0.001	75 (57–82)	<0.001	76 (62–86)	<0.001
No	84	22 (17–31)		86 (64–96)		90 (71–100)	

^a^P-values derived using Wilcoxon Rank Sum (for 2 sample comparisons) or Kruskal Wallis (for 3 sample comparisons) tests

^b^Derived from Impact on Family Scale (Stein, et al. [27]). Higher score indicates *higher* impact. Impact on Family Scale (range of possible scores, 0 to 56)

^c^Derived from Infant Toddler Quality of Life (ITQOL) questionnaire (Landgraf, et al. [30]). Higher score indicates *lower* impact. ITQOL scale (range of possible scores, 0 to 100)

^d^Use of one or more public assistance programs: Supplemental nutritional assistance program for Women, Infants and Children, Transitional Aid to Families with Dependent Children, Low Income Home Energy Assistance Program

^e^Unsafe home environment constitutes water leaks, pests, or no heat

^f^Social isolation defined by positive response to query about feelings of isolation

### Adjusted associations of potentially modifiable characteristics with outcomes

Table 3 shows associations of potentially modifiable characteristics with the total IOF Scale scores, adjusting for potential confounders. Taking time off from work without pay, increased bills, financial worry, an unsafe home environment, and social isolation were all associated with higher total IOF scores, indicating greater impact. Similarly, Table 4 shows adjusted associations of potentially modifiable characteristics with impact on Parent Emotion and Time Limitation scores. Taking time off from work without pay and social isolation were associated with lower scores on both of these scales, indicating greater impact. Financial worry was associated with greater impact on parent time limitation, as was an unsafe home environment. In contrast, enrollment in early intervention and Medicaid programs were associated with **higher** parent time limitation scores, indicating **less** parental impact.

**Table 3:** Adjusted associations of potentially modifiable characteristics with total impact on family scores (*n* = 196)

Characteristics	Difference in impact on family scale (95% confidence interval)^a^	*p*-value
*Use of community based resources*		
Enrollment in early intervention	−1 (−8, 6.7)	0.8
Use of social services^c^	5.7 (0.2, 11)	0.04
Use of public housing	0.3 (−9.5, 10.1)	0.9
Receive supplemental security income	−2.8 (−8.2, 3.2)	0.4
Use of a community based clinic for primary care	−2.3 (−8.8, 4.1)	0.5
*Financial burden since infant discharge*		
Family member has taken ≥ 3 h/week off from work without pay	4.7 (0.8, 8.7)	0.02
Family member has received compensation by employer for time off	1.3 (−2.9, 5.6)	0.5
No compensation (from any source) for time off	4.8 (−0.3, 9.9)	0.06
Financial burden		
Unexpected costs	1.5 (−2.8, 5.7)	0.7
Increased bills	6.1 (0.4, 11.9)	0.04
Increased out-of-pocket expenses	−1.5 (−7, 4.5)	0.6
Financial worry	4 (0.2,7)	0.04
Collections discussed prior to discharge	2.5 (−2.5, 7)	0.4
Enrollment in medi-caid	1.3 (−3.5, 6)	0.6
*Health related social problems*		
Unsafe home environment^d^	6.3 (0.2, 12.3)	0.04
Social isolation^e^	8.5 (4, 12.9)	0.0003

Models adjusted for: infant birthweight (per 500 g increase), race, family annual household income, infant co-morbidities during hospitalization in NICU (including of the following: growth restriction, surfactant deficiency, necrotizing enterocolitis, intraventricular hemorrhage grade 3 or 4, patent ductus arteriosus, retinopathy of prematurity)

^a^Estimates represent differences in points on Impact on Family Scale (range of possible scores, 0 to 56). Higher scores represent higher impact

^c^Use of one or more public assistance programs: Supplemental nutritional assistance program for Women, Infants and Children, Transitional Aid to Families with Dependent Children, Low Income Home Energy Assistance Program

^d^Unsafe home environment constitutes water leaks, pests, or no heat

^e^Social isolation defined by positive response to query about feelings of isolation

**Table 4:** Adjusted associations of potentially modifiable characteristics with impact on parent emotion and time limitation scores (*n* = 196)^a^

Characteristics	Total (*n* = 196)^b^	
	Parent emotion points (95% confidence interval)	*p*-value
*Use of community based resources*		
Enrollment in early intervention	4.2 (−12, 20.5)	0.6
Use of social services^c^	−2.3 (−14, 9.5)	0.7
Use of public housing	7.9 (−13, 29)	0.5
Receive supplemental security income	0.5 (−12, 13.3)	0.9
Use of a community based clinic for primary care	1.9 (−12.3, 16)	0.8
*Financial burden since infant discharge*		
Family member has taken ≥ 3 h/week off from work without pay	−12.4 (−20, −4)	0.004
Family member has received compensation by employer for time off	4.5 (−4.3, 14)	0.3
No compensation (from any source) for time off	−5.2 (−16, 5)	0.4
Financial burden		
Unexpected costs	−2.7 (−11, 6)	0.6
Increased bills	−5 (−17, 7)	0.4
Increased out-of-pocket expenses	2.5 (−9, 14)	0.4
Financial worry	−4.7 (−13, 3)	0.3
Collections discussed prior to discharge	1.1 (−9, 12)	0.8
Enrollment in medi-caid	4.9 (−5, 15)	0.4
*Health related social problems*		
Unsafe home environment^d^	−1.2 (−14, 12)	0.8
Social isolation^e^	−11.2 (−21, −1)	0.03
Characteristics	Total (n = 196)	
	Parent limitation on time score points (95% Confidence Interval)	*p*-value
*Use of community based resources*		
Enrollment in early intervention	20.2 (5.6, 34)	0.007
Use of social services^c^	1.9 (−8.7, 12.5)	0.7
Use of public housing	3.5 (−15, 22)	0.7
Receive supplemental security income	0.1 (−15, 22)	0.7
Use of a community based clinic for primary care	5.5 (−7, 18)	0.4
*Financial burden since infant discharge*		
Family member has taken ≥ 3 h/week off from work without pay	−8.8 (−16.9, −1)	0.03
Family member has received compensation by employer for time off	5.4 (−3.5, 15)	0.2
No compensation (from any source) for time off	−10.3 (−21, 0.5)	0.07
Financial burden		
Unexpected costs	−1 (−9, 9)	0.9
Increased bills	−4.9 (−17, 7.4)	0.4
Increased out-of-pocket expenses	3.2 (−8, 15)	0.6
Financial worry	−10.4 (−18, −2)	0.01
Collections discussed prior to discharge	−6 (−17, 4.7)	0.3
Enrollment in medi-caid	12.6 (2.6, 22)	0.01
*Health related social problems*		
Unsafe home environment^d^	−13.2 (−26, −1)	0.04
Social isolation^e^	−11.4 (−21, −2)	0.02

^a^Estimates represent differences in points on Infant and Toddler Life Questionnaire (range of possible scores, 0 to 100). Lower scores represent higher impact

^b^Models adjusted for: infant birthweight (per 500 g increase), race, family annual household income, infant co-morbidities during hospitalization in NICU (including: growth restriction, surfactant deficiency, necrotizing enterocolitis, intraventricular hemorrhage grade 3 or 4, patent ductus arteriosus, retinopathy of prematurity)

^c^Use of one or more public assistance programs: Supplemental nutritional assistance program for Women, Infants and Children, Transitional Aid to Families with Dependent Children, Low Income Home Energy Assistance Program

^d^Unsafe home environment constitutes water leaks, pests, or no heat

^e^Social isolation defined by positive response to query about feelings of isolation

## Discussion

In this study, we described the impact of preterm birth on parents and families in the first 2 years after neonatal discharge. Our results support our conceptual model, which posits modifiable factors that are associated with the impact of preterm birth on parents and families, **independent** of infant health and socio-demographic characteristics. We identified several potentially modifiable factors that were associated with both higher and lower impact. In particular, social isolation, financial burdens such as taking unpaid time off from work, increased bills and financial worry, and an unsafe home environment were all associated with higher impact on at least one of our main outcomes. In contrast, enrollment in early intervention and Medicaid and use of public housing were associated with less parent impact.

Predisposing characteristics such as infant co-morbidities affected both impact on family scores and parental scores. Infant development affected parental scores for increased anxiety and emotion. Our findings were consistent with previous studies that the impact was greater among families whose preterm children demonstrated either a functional handicap or low developmental quotient [22, 25, 57–59]. We also found that the use of prescription medications and durable medical equipment affected both parental impact scores and impact on family scores, which was consistent with other publications [20, 60]. Specifically, the use of medications and medical equipment may contribute to the substantial out-of-pocket expenditures that families may incur [61, 62]. As pressure mounts to reduce hospital length of stay and readmission rates, and as we move more complex care into the community, high out-of-pocket costs is an important factor that can contribute to parental and family strain.

Several studies have shown that preterm birth and an infant’s hospitalization can adversely affect the finances of families after the birth of a preterm or VLBW infant [20, 60–63]. However, little is known about a more modifiable determinant such as the specifics of financial burden faced by families, or about the impact of financial burden on parent quality of life. In our study, we found that a lack of compensation for time off work was associated with both family and parent-time impact scores. Also, increased bills due to hospitalization and increased financial worry were associated with greater impact. Complementing our findings, 2 studies have reported the out-of-pocket costs incurred by families of preterm infants for outpatient services, medications, as well as indirect costs like lost productivity are significant especially during the first year after discharge [60, 64]. Specifically, Hodek et al. cited that co-payments for outpatient ancillary services and medications increased parental out-of-pocket expenses. Moreover, lost wages for missing work days may increase income losses [60]. Overall, by highlighting the specific aspects of financial burden most closely associated with parent and family impact, such as lack of compensation and increased bills, our results may inform targeted financial support programs for families of preterm infants after discharge. Moreover, our findings support that while annual income was not associated with impact on family, parental perspective on financial burden was, which should also be considered when caring for these families.

Another modifiable determinant are health related social problems. These are economic and social problems that can affect health such as food insecurity and substandard housing [45]. Prior studies have demonstrated the impact of substandard housing on child health such as increased infectious disease and injury [65]. However, we found that an unsafe home environment was associated with adverse parent-time impact and impact on the family. Timely receipt of public housing has been associated with improved health in other medical condtions [66]. Addressing housing concerns for families of preterm infants through existing public housing programs is a feasible approach to reducing the parental and family impact.

Another health related social problem that was associated with greater parental *and* family impact was social isolation. Other studies that have examined families of premature infants have found that “alienation” [67] and social isolation may have profound impact on parental emotion [68]. Jackson et al. described the paradigm of the process of acclimatization of caring for a premature infant as alienation, responsibility, confidence and familiarity, and that alienation may be protracted in this population [67]. Intervention strategies that have improved parental emotions often include education-behavioral models. For example, the Creating Opportunities for Parent Empowerment (COPE) program created by Melnyk, et al. was associated with reduced parental impact during transition home from the NICU [69, 70]. It is possible that programs like that one benefit families by reducing social isolation in months to years after discharge.

Another modifiable determinant is enrollment in a community-based developmental program like “Early Intervention (EI),” was associated with less impact on parental limitation of time. A recent meta-analysis suggests that community-based developmental programs had beneficial pooled effects on maternal anxiety, depressive symptoms, and self-efficacy [71]. Moreover, other studies have suggested that these programs can also empower families because of the collaborative process that EI offers; in turn, they have a deeper understanding of their child’s developmental needs [72].

Similarly to early intervention, we found that receipt of Medicaid was associated with lower impact scores on limitations on time. Other studies have demonstrated families who were registered with Medicaid showed improved “parent role confidence” and “parent-baby interaction” than those with private insurance [69]. While this result was unexpected, it has been speculated that parents with a higher socioeconomic status and private insurance may have higher expectations for themselves [69] and therefore may perceive an increased parental impact on their time versus those who utilize Medicaid. Overall, our results suggest that greater participation in public assistance programs may lessen familial and parental burden for this patient population.

A strength of our study was our high response rate (82%). Characteristics of the infants were very similar to other follow-up programs [25]. However, we studied families of infants presenting for follow-up care rather than the underlying population of families of infants receiving neonatal intensive care, potentially limiting generalizability. Moreover, we did sample both mothers *and* fathers, which may affect how some of our results are interpreted and parent gender may influence some of the measures including financial burden and social isolation [73]. Also, our study was cross-sectional making us unable to establish causation. While we adjusted for a number of potential confounders, like all observational studies, ours is subject to residual confounding. While we did not have a full term cohort control in comparison [25], nor data on those not enrolled, our aim was to elicit the experience of parents and families of preterm infants, as well as relationships with modifiable characteristics specific to this population.

## Conclusions

In summary, we identified several predictors of increased family and parent impact in families of preterm infants. Of particular interest were the potentially modifiable factors including social isolation and financial burden, which were associated with greater impact, and use of community-based developmental services, public housing, and Medicaid which were associated with less impact. Our results suggest that interventions to target these factors, for example social and financial support programs, and efforts to increase enrollment in community-based developmental services and public health insurance programs, might lessen the impact of preterm birth on parents and families.

## Additional files

Additional file 1: Presurvey information demographics. (DOC 108 kb)
Additional file 2: Table S1. Description of measurement instruments for primary outcomes, modifiable determinants and predisposing characteristics (potential confounders). (DOC 55 kb)

## Abbreviations

IOF: Impact on family total score
ITQOL: Infant toddler quality of life
MSD: Motor and social development
NICU: Neonatal intensive care unit
PFB: Perceived financial burden
