# Supplementary material for: The impact of preterm birth <37 weeks on parents and families: a cross-sectional study in the 2 years after discharge from the neonatal intensive care unit
Source: Health Qual Life Outcomes. 2017 Feb 16;15:38. doi: 10.1186/s12955-017-0602-3 (PMC5312577; doi:10.1186/s12955-017-0602-3)
Supplement: Additional file 1: — Presurvey information demographics. (DOC 108 kb) [file 12955_2017_602_MOESM1_ESM.doc]

**PRESURVEY INFORMATION**

**DEMOGRAPHICS**

A. Respondent Contact Information

1. Respondent's email address

Please type in response

2. Main phone number:

Please type in response

3. Name:

Please type in response

4. Address:

Please type in response

B. Child’s Information

1. Child’s Name🡪REFER TO AS[ CHILD’S NAME]

1. Type in Response

2. Medical Record Number

1. Type in Response

🡪ASSIGN SUBJECT ID

3. Child’s Sex

1. Male

2. Female

3. Medical Record Information

1. Date of Visit🡪Enter today’s date and time

2. Mother’s age

3. Gravida Status

4. Parity Status

5. Complications of pregnancy

1. Pregnancy induced hypertension

2. Preeclampsia

3. Chorioamniotitis

4. IUGR/SGA
5. Gestational Diabetes Mellitus

6. Preterm Labor

7. Other

🡪TEXT BOX IF OTHER

6. Type of Delivery

1. Vaginal

2. Cesarean

3. Forceps

4. Vacuum

7. Hospital of Delivery

1. Brigham and Women’s

2. Beth Israel Deaconess

3. Mass General

4. Boston Medical Center

5. Other

🡪TEXT BOX IF OTHER

8. Infant Demographics

1. Birthweight

1. Type in Response

2. Gestational Age

1. Type in Response, Weeks/Days

3. Multiple Gestation

1. Singleton

2. Twin

3. Triplet or higher

4. Length of hospitalization

1. Type in Response

🡪PLEASE CALCULATE CORRECTED AGE IN WEEKS_____DAYS_____

9. Comorbidities

1. CPAP

2. Intubated

3. Surfactant Deficiency or RDS

4. Medical NEC

5. Surgical NEC

6. IVH

7. PDA s/p Ligation

8. PDA s/p Indomethacin

9. Other

Type in Response

I. DEMOGRAPHICS/CONSENT

Thank you for participating in this survey. By checking the following, you are providing your consent to participate in the study “Quality of Life among caregivers and families of preterm infants after discharge from the NICU.”

1. Yes

2. No

2. Are you the baby’s:

1. Mother
2. Father
3. Other

🡪[IF I.2 is Response 3]

7.If other, please type in your relationship with the baby.

1. Please type in response🡪APPEAR TEXT BOX

**II. Transition from the NICU to the ambulatory care setting**

**The next set of statements is about the time you were taking your baby home from the hospital. Please answer with the following responses. Please answer each of the questions with one of the following responses.**

1. Strongly agree
2. Disagree
3. Agree
4. Strongly agree
5. Don’t know/don’t remember/not applicable

1. Before [CHILD’S NAME] left the hospital, the staff and I agreed about clear health goals for [CHILD’S NAME] and how these would be reached.

2. The hospital staff took my preferences and those of my family into account in deciding what the health care needs would be for [CHILD’s NAME] when we left the hospital.

3. The hospital staff took my preferences and those of my family into account in deciding where my baby’s health care needs would be met when we left the hospital.

4. When [CHILD’S NAME} left the hospital, we had all the information we needed to be able to take care of [CHILD’S NAME].

5. When we left the hospital, we clearly understood how to manage the baby’s health.

6. When we left the hospital, we clearly understood the warning signs and symptoms we should watch for to monitor [CHILD’S NAME].

7. When we left the hospital, we had a readable and easily understood written plan that described how all of [CHILD’S NAME]’s health care needs were going to be met.

8. When we left the hospital, we had a good understanding of my baby’s health condition and what makes it better or worse.

9. When we left the hospital, we had a good understanding of the things we were responsible for in managing [CHILD’S NAME]’s health.

10. When we left the hospital, we were confident that we knew what to do to manage [CHILD’S NAME]’s health.

11. When we left the hospital, we were confident that we could actually do things we needed to do to take care of [CHILD’S NAME]’s health.

**The next statement is about your follow up doctors’ appointments and baby’s medications.**

12. When we left the hospital, we had a readable and easily understood written list of the appointments or tests we needed to complete within the next several weeks.

13. When we left the hospital, we clearly understood the purpose for each of [CHILD’S NAME]’s medications.

14. When we left the hospital, we clearly understood how to administer each of [CHILD’S NAME]’s medications including how much we should give and when.

15. When we left the hospital, we clearly understood the possible side effects of each of [CHILD’S NAME]’s medications.

**III. POST DISCHARGE: INFANT CARE. The next set of questions are about [CHILD’S NAME]’s general health in the past month.**

1. GENERAL HEALTH

1. Do you have a primary place for the baby’s medical care?

1. Yes
2. No

🡪[IF RESPONSE to question IIIA.1 is YES]

2. Is this place for medical care a:

1. Private clinic/medical group?

2. Hospital clinic?

3. Neighborhood Health Center?

4. City or County public health center?

🡪[IF RESPONSE to question IIIA.2 is NO]🡪SKIP

3. Since [CHILD’S NAME] came home for the first time, were there any other times until now that [CHILD’s NAME} had to stay in the hospital?

1. Yes
2. No

🡪[IF RESPONSE TO QUESTION IIIA.3 is RESPONSE=2. NO, SKIP TO 5]

[IF RESPONSE TO QUESTION IIIA.3 is Response 1=YES]

4. If so how many times?
1. 1

2. 2

3. 3

4. 4

5. More than 4 times

[IF RESPONSE TO QUESTION IIIA.3 is RESPONSE =1=YES]

Was [CHILD’s NAME] hospitalized for any of the following reasons. Please check all that apply.

1. Breathing problems (Your doctors might have called this ‘RSV infection’ or ‘bronchiolitis’)

2. Dehydration

3. Feeding problems

4. Infection

5. Apnea (times where your child stopped breathing

6. Injury

7. Other breathing problems

8. Poor weight gain

9. Other

[IF RESPONSE IS 9OTHER]🡪APPEAR TEXT BOX TO TYPE IN RESPONSE

5. Has your baby had any surgery or operations since coming home from the hospital?

1. Yes
2. No

🡪[IF RESPONSE TO QUESTION IIIA.5 is RESPONSE=2. NO, SKIP TO 8]

[IF RESPONSE TO QUESTION IIIA.5 is Response 1=YES], SKIP TO QUESTION 6

[IF RESPONSE TO QUESTION IIIA.5 is Response 1=YES]

6. If so, please describe the operation. Please check all that apply.

1. Gastrostomy tube or feeding tube placement
2. Hernia repair
3. Eye surgery
4. Other

[IF RESPONSE TO QUESTION IIIA.6 is RESPONSE 4=OTHER]

7. If you responded other, please type in response.

1. Type in Response🡪APPEAR TEXT BOX

8. Has {CHILD’s NAME] had any accidents or injuries or illnesses and seen in the emergency room since coming home from the NICU?

1. Yes
2. No

[IF RESPONSE TO QUESTION IIIA.8 is NO=2=SKIP TO QUESTION 11]

[IF RESPONSE TO QUESTION IIIA.8 is RESPONSE=1=YES]

9. If so, please describe these illnesses. Please check all that apply

1. Breathing problems (Your doctors might have called this ‘RSV infection’ or ‘bronchiolitis’)

2. Dehydration

3. Feeding problems

4. Infection

5. Apnea (times where your child stopped breathing

6. Injury

7. Other breathing problems

8. Poor weight gain

9. Other

[IF RESPONSE IS 9 OTHER]🡪APPEAR TEXT BOX TO TYPE IN RESPONSE

[IF RESPONSE TO QUESTION IIIA.9 is RESPONSE=9=OTHER]

10. If you responded, other, please type in response.

1. Type in response🡪APPEAR TEXT BOX

15. Does your baby require dependence on technology? Please check all that apply.

1. Oxygen
2. Tracheostomy or breathing tube
3. Feeding Tube
4. Wheelchair
5. Adaptive Stroller
6. Other
7. None of the above

[IF RESPONSE TO QUESTION IIIA.15 WAS 6=OTHER]

16. If you responded other, please type in response.

1. Type in response

17. Do you give [CHILD’S NAME] any medications?

1. Yes

2. No

[IF RESPONSE IS 2=NO, SKIP to BEHAVIORS/DEVELOPMENT]

18. [IF RESPONSE=1=YES] Please describe these medications. Please check all that apply.

- - 1. Iron supplementation
    2. Vitamin D
    3. Diuretics like “LASIX” OR “DIURIL”
    4. Multivitamins
    5. Antibiotic prophylaxis
    6. OTHER

19. [IF OTHER=6], Please describe medications🡪APPEAR TEXT BOX

1. BEHAVIORS/DEVELOPMENT. The following are questions about your baby’s development. Please answer each check all that apply.

**The following questions are adapted from the MSD Scale and will incorporate skip logic to the corrected age of the patient.*

****Provided example for 0-3 months, other age categories are included in the appendix***

If the calculated age is between 0-3 months:

1. When lying on (his/her) stomach, has your child ever turned his/her head from side to side?

2. Has your child’s eyes ever followed a moving object?

3. When lying on his/her stomach on a flat surface, has your child ever lifted his/her head off the surface for a moment?

4. Has your child ever following a moving object all the way from one side to another?

5. Has your child ever smiled at someone when that person talked to or smiled at (but did not touch him/her)?

6. When lying on his/her stomach, has your child ever raised his/her head and chest from the surface while resting his/her weight on his/her lower arms or hands?

7. Has your child even turned his/her head around to look at something?

8. While lying on his/her back and being pulled up to a sitting position, did your child even hold his/her head stiffly so that it did not hang back as h/she was pulled up?

9. Has he/she ever laughed out loud without being tickled or touched?

10. Has your child ever held in one hand a moderate sized object such as a block or rattle?

11. Has your child ever rolled over on his/her own on purpose?

12. Has he/she ever seemed to enjoy looking in the mirror at him/herself?

13. Has your child ever been pulled from a sitting to standing position and supported his/her own weight with legs stretched out?

14. Has your child ever looked around with his/her eyes for a toy which was lost or not nearby?

15. Has your child ever sat alone with no help except for leaning forward on his/her hands with just a little help from someone else?

1. SCREENING/FOLLOW UP. The next few questions ask about other services your baby may need.

1. Has [CHILD’S NAME] seen any medical specialists since being home?

1. Yes
2. No

[IF RESPONSE TO QUESTION IIIC.2 IS NO=2, SKIP TO QUESTION 4]

[IF RESPONSE TO QUESTION IIIC.1 IS YES=1]

2. If so, please check all that apply.

1. Neurology
2. Orthopedics
3. Gastrointestinal
4. Endocrinology
5. Pulmonary
6. Other

[IF RESPONSE TO QUESTION IIIC.2 WAS 6=OTHER}

3. If you responded other, please type in response.

1. Type in response🡪APPEAR TEXT BOX

4. How many medical appointments has your child had in the past one month?

1. 1
2. 2
3. 3
4. 4
5. 5
6. More than 5

5. Were you ever informed by caretakers or your pediatrician in the NICU about Early Intervention services before [CHILD’S NAME} was discharged home?

1. Yes
2. No

6. Did your pediatrician or caretakers in the NICU recommend that [CHILD’s NAME] receive Early Intervention Services?
1. Yes

2. No

6. Were you able to obtain these services?

1. Yes
2. No

7. [IF RESPONSE=2=NO], Please check all that apply as to why you did not obtain Early Intervention services.

1. Difficulty with transportation

2. [CHILD’S NAME] did not qualify for services

3. You decided it wasn’t necessary

4. Other

[IF OTHER=4]Please type in response🡪APPEAR TEXT BOX

8. Which of the following specialists do you receive services from and do you receive services as described? Please check all that apply.

1. Home Registered Nurse
2. Visiting Nurse
3. Licensed practical nurse
4. Nurse’s aide
5. Physical therapist
6. Occupational therapist
7. Speech therapist
8. Developmental educator
9. Feeding specialist
10. Social worker
11. Other

[IF OTHER=10, Please type in response🡪APPEAR TEXT BOX]

IV. FAMILY QUALITY OF LIFE AND CAREGIVER PHYSICAL/MENTAL HEALTH.

**The next set of questions will help us know you feel and how well you (NOT your baby) are.**

If you are unsure about how to answer a question, please give the best

answer you can and make a written comment beside your answer.

1. In general, would you say your health is:

Excellent (1)

Very Good (2)

Good (3)

Fair (4)

Poor (5)

The following two questions are about activities you might do during a typical day. Does YOUR

HEALTH NOW LIMIT YOU in these activities? If so, how much?

2. MODERATE ACTIVITIES, such as moving a table, pushing a vacuum cleaner, bowling, or playing

golf:

Yes, Limited A Lot (1)

Yes, Limited A Little (2)

No, Not Limited At All (3)

3. Climbing SEVERAL flights of stairs:

Yes, Limited A Lot (1)

Yes, Limited A Little (2)

No, Not Limited At All (3)

During the PAST 4 WEEKS have you had any of the following problems with your work or other regular

activities AS A RESULT OF YOUR PHYSICAL HEALTH?

4. ACCOMPLISHED LESS than you would like:

Yes (1)

No (2)

5. Were limited in the KIND of work or other activities:

Yes (1)

No (2)

During the PAST 4 WEEKS, were you limited in the kind of work you do or other regular activities AS A

RESULT OF ANY EMOTIONAL PROBLEMS (such as feeling depressed or anxious)?

6. ACCOMPLISHED LESS than you would like:

Yes (1)

No (2)

7. Didn’t do work or other activities as CAREFULLY as usual:

Yes (1)

No (2)

8. During the PAST 4 WEEKS, how much did PAIN interfere with your normal work (including both work

outside the home and housework)?

Not At All (1)

A Little Bit (2)

Moderately (3)

Quite A Bit (4)

Extremely (5)

The next three questions are about how you feel and how things have been DURING THE PAST 4

WEEKS. For each question, please give the one answer that comes closest to the way you have been

feeling. How much of the time during the PAST 4 WEEKS –

9. Have you felt calm and peaceful?

All of the Time (1)

Most of the Time (2)

A Good Bit of the Time (3)

Some of the Time (4)

A Little of the Time (5)

None of the Time (6)

10. Did you have a lot of energy?

All of the Time (1)

Most of the Time (2)

A Good Bit of the Time (3)

Some of the Time (4)

A Little of the Time (5)

None of the Time (6)

11. Have you felt downhearted and blue?

All of the Time (1)

Most of the Time (2)

A Good Bit of the Time (3)

Some of the Time (4)

A Little of the Time (5)

None of the Time (6)

12. During the PAST 4 WEEKS, how much of the time has your PHYSICAL HEALTH OR EMOTIONAL

PROBLEMS interfered with your social activities (like visiting with friends, relatives, etc.)?

All of the Time (1)

Most of the Time (2)

A Good Bit of the Time (3)

Some of the Time (4)

A Little of the Time (5)

None of the Time (6)

1. Family Quality of Life*

The following questions are about your child’s impact on you

During the past 4 weeks, how much anxiety or worry did each of the following cause You?

Please answer with one of the following:

None at all

A little bit

Some

Quite a bit

A lot

1. Your child’s feeding/eating/sleeping habits

2. Your child’s physical health

3. Your child’s emotional well being

4. Your child’s learning abilities or cognitive development

5. Your child’s ability to interact with others

6. Your child’s behavior

7. Your child’s temperament

Please answer the next set of questions with the following responses

Yes, limited a lot

Yes, limited some

Yes, limited a little

Not limited

1. Feeding/eating/sleeping habits

2. Physical health

3. Emotional well being

4. Learning or cognitive development

5. Ability to interact with others

6. Behavior

7. Temperament

Sometimes families may have difficulty getting along with one another. They do not always agree and they may get angry. In general, how would you rate your family’s ability to get along with one another?

Excellent

Very Good

Good

Fair

Poor

**Please answer the following questions as:**

**1. Strongly agree**

**2. Somewhat Agree**

**3. Somewhat Disagree**

**4. Strongly disagree**

**5. Refuse**

IF1. Additional income is needed in order to cover medical

expenses.

IF2. The illness is causing financial problems for the family **n**

IF3. Time is lost from work because of hospital appointments **n**

IF4. I am cutting down the hours I work to care for my child. **n**

IF5. Our family gives up things because of my child's illness. **n**

IF6. People in the neighborhood treat us specially because of

my child's illness.

IF7. We see family and friends less because of the illness. **n**

IF8. I don't have much time left over for other family members

after caring for my child.

IF9. We have little desire to go out because of my child's illness. **n**

IF10. Because of the illness we are not able to travel away from

Home.

IF11. Sometimes we have to change plans about going out at the

last minute because of my child's illness.

IF12. Sometimes I wonder whether my child should be treated

"specially" or the same as a normal child.

IF13. I think about not having more children because of the

illness.

IF14. Nobody understands the burden I carry.

IF15. Traveling to the hospital is a strain on me. **n**

IF16. Sometimes I feel like we live on a roller coaster - in crisis

when my child is acutely ill and OK when things are more stable.

IF17. It is hard to find a reliable person to take care of my child. **n**

IF18. I live from day to day and don't plan for the future. **n**

IF19. Fatigue is a problem because of my child's illness.

IF20. Learning to manage my child's illness has made me feel

better about myself.

IF21. Because of what we have shared we are a closer family **n**

IF22. My partner and I discuss my child's problems together. **n**

IF23. We try to treat my child as if he/she were a normal child.

IF24. My relatives have been understanding and helpful with my

child.

V. SOCIODEMOGRAPHIC DETERMINANTS AND HEALTH CARE COSTS

A. The next set of questions asks you about health care costs related to your baby’s health.

Please check the category which shows the family’s income?

1. <$20,000 annually
2. 20,001-40,000
3. 40,001-60,000
4. 60,001-80,000
5. Greater than 80,000

1. Do you work?

1. Yes

2. No

[IF RESPONSE TO QUESTION VA. IS NO=2, SKIP TO QUESTION 5]

[IF RESPONSE TO QUESTION VA IS YES=1]

1. Have you taken time off work because of the baby’s birth?

1. Yes

2. No

[IF RESPONSE TO QUESTION VA.1 is 1=YES]

2. If yes, how many weeks in the past 4 weeks?

1. 1

2. 2

3. 3

4. 4

[IF RESPONSE TO QUESTION VA.1 is 1=YES]

3. Is the time taken off work by you considered:

1. Sick leave, maternity/paternity leave or personal time paid by your employer
2. Maternity/paternity leave paid by government
3. Vacation
4. Time off with no pay
5. I am self-employed
6. Don’t Know

7. Unemployment

4. Please tell us your individual salary (note: all your responses are completely confidential):

1. Please type in response (dollars/per year)🡪APPEAR TEXT BOX

5. Does your partner/spouse work?

1. Yes

2. No

3. Unemployed

4. Not applicable

[IF RESPONSE NO=2, SKIP TO QUESTION 9]

[IF YES, PROCEED TO QUESTION 6]

1. Has your partner/spouse taken time off from work because of the baby’s birth?

[IF RESPONSE TO QUESTION VA.5 is RESPONSE=1, YES]

6. If yes, how many weeks in the past 4 weeks?

1. 1

2. 2

3. 3

4. 4

[IF RESPONSE TO QUESTION VA.5 is 1=YES]

7. Is the time taken off work by your partner/spouse considered:

1. Sick leave, maternity/paternity leave or personal time paid by your employer

2. Maternity/paternity leave paid by government

3. Vacation

4. Time off with no pay

5. I am self-employed

6. Don’t know

7. Unemployment

8. Please tell us your partner’s salary (note: all your responses are completely confidential):

1. Please type in response (dollars/year)🡪APPEAR TEXT BOX

9. Sometimes family members or friends take time off work to help a mother with her new baby. Did your family members or friends (other than your husband/partner) take time off work to help you/your family?

1. Yes

2. No

[IF NO, SKIP TO QUESTION 12]

[IF YES, PLEASE PROCEED TO QUESTION 10]

10. If yes, how many weeks in the past 4 weeks?

1. 1

2. 2

3. 3

4. 4

[IF RESPOSNE TO QUESTION VA.9 is RESPONSE=1, YES}

11. Was the time taken off work by your family member or friend considered:

1. Sick leave, maternity/paternity leave or personal time paid by your employer
2. Maternity/paternity leave paid by government
3. Vacation
4. Time off with no pay
5. My family member or friend is self-employed
6. Don’t know
7. Unemployment

12. Have you received household help (housekeeping, cleaning, etc) that you paid for?

1. Yes

2. No

[RESPONSE TO QUESTION VA.12 IS 1=YES]

13.If yes, what was the cost per week?

1. Dollars/weeks, Type in response

14.Do you travel to the hospital for your baby’s appointments, other than by walking?

1. Yes
2. No

[IF QUESTION VA.14 is 1=YES]

15. If yes, what is the number of round trips you have made in the past 4 weeks?

1. 1
2. 2.
3. 3
4. 4
5. >4

16. If yes, how do you travel to your appointments, please check all that apply.

1. Car

2. Public Transit (Train/Bus)

3. Taxi

16. If yes, and you travel by car, what is the cost of parking for each trip?

1. $10
2. $11-15
3. $16-20
4. >$20

17. If you travel by public transit, what is the cost per trip (each direction)?

1. <$5
2. $6-10
3. $11-15
4. >$15

18. If yes and you travel by taxi, what is the cost per trip in the past 4 weeks?

1. <$10
2. $11-20
3. $21-30
4. >$30

19. Have you hired caretakers or babysitters for your other children in order for you to allow for follow up care for your baby?

1. Yes
2. No

[IF QUESTION VA.19 is 1=YES]

20.If yes, how many hours per week?

1. 1
2. 2
3. 3
4. 4
5. >4

21. What is the cost/week for childcare?

1. <$50
2. 51-100
3. 101-150
4. 151-200
5. >$200/week

21. Have you used a breast pump in the past 4 weeks?

1. Yes
2. No

[IF QUESTION VA.21 is 1=YES]

If yes, how was this paid for? Please choose one.

1. Rented by you
2. Purchased by you
3. Loaned by hospital
4. Paid for or reimbursed fully by insurance

[If you rented or purchased, RESPONSES 1 or 2], what was the cost to you?

1. Type in response🡪APPEAR TEXT BOX

The next set of questions asks you about your family’s healthcare costs

1. In the past 12 months, did any unexpected events happen that caused your family’s medical costs to be higher than you had planned?

1. Yes

2. No

2. During the last 12 months, were there times when you had problems paying or were unable to pay at all for medical bills?

1. Yes

2. No

3.Did your out of pocket expense costs in your health care plan end up being as you expected?

1. Yes

2. No actual costs were higher

3. No actual costs were lower

4.During the last 12 months, have you had to do any of the following because of your medical costs?

1. Borrow money or increase the use of your credit cards

2. Use any of your savings

3. Set up a payment plan with your hospital or doctor’s office

4. Had a bill sent to collections

6. Did you make any other financial adjustments?

1. Yes

2. No

7.Did you discuss the costs that you would have had to pay for your child’s health care with your child’s doctor?

1. Yes

2. No

8.During the past 12 months how often did your worry about the cost of health care for your family?

1. Never

2. Sometimes

3. Very Often

4. Frequently

9.When you were in the NICU, did anyone discuss SSI (Supplemental Security Income) benefits with your family?

1. Yes

2. No

10. If so, are you receiving SSI benefits for your child?

1. Yes

2. No

11. How much income did you receive in the past 12 months, per month?

1. 500/month

2. 500-1000/month

3. 1000-1500

4. 1500-2000

5. 2000-2500

6. 2500-3000

7. Greater than 3000/month

III. SOCIODEMOGRAPHIC DETERMINANTS OF ACCESS TO HEALTHCARE*

Does [CHILD’S NAME] lives with the:

1. Mother
2. Father

If the child does not live with mother/father, how often does he see them?

1. Daily
2. Almost daily
3. Once a week
4. 2-3 times/month
5. Rarely
6. Never

The child’s parents are currently:

1. Married
2. Separated
3. Divorced
4. Never married

What is the highest number of years of school completed by the baby’s biological mother?

1. Less than high school
2. High School
3. Some college
4. Bachelor’s degree
5. Graduate degree

What is the highest number of years of school completed by the baby’s biological father?

1. Less than high school
2. High School
3. Some college
4. Bachelor’s degree
5. Graduate degree

What is the mother’s occupation?

1. Type in response

What is father’s occupation?

1. Type in response

What languages are spoken in the household most of the time?

1. English
2. Spanish
3. Other
4. If other, type in response

Does anyone in the household smoke cigarettes?

1. Yes
2. No

How would you describe your race? Please check all that apply. You may click on the checkbox or on the words next to it.

1. Black or African-American

2. Asian or Pacific Islander

3. American Indian or Native Alaskan

4. White

5. Other

Was there a time in the past 12 months when you or anyone in your household needed

medical care or surgery, but could not get it?

1. Yes

2. No

3. Not Sure

What is the main reason someone in your household could not get medical care or

surgery? If this has happened more than once, please select the most recent reason.

1. Cost

2. No insurance

3. No childcare

4. Distance

5. Office hours did not work for you / Could not miss school or work

6. No transportation

7. Too long a wait for an appointment

8. Other

In the past 12 months, was there any time when you could not fill prescriptions for

medicine?

1. Yes

2. No

The next few questions are about your current household. What best describes your current living situation?

1. Rent apartment

2. Rent room(s) within another person's home

3. Own home

4. Homeless or living in a shelter

5. Doubled up with another family

6. Live with parents/other family

7. Other

Do you have a safe place to stay tonight?

1. Yes

2. No

3. Not sure

Including yourself, how many adults and children that are related to you live in your

household? A household includes your spouse/partner and other family members that live with you. Non-relatives, such as housemates, do not count.

1. 1

2. 2

3. 3

4. 4

5. 5

6. 6

7. 7

8. 8 or more

You said 8 or more adults and children live in your household. To be specific, how many?

1. 8

2. 9

3. 10

4. 11

5. 12

6. 13

7. 14

8. 15 or more

Are you on a waiting list for public or subsidized housing?

1. Yes

2. No

3. Not sure

In the last 12 months has the electric or gas company threatened to shut off or shut off the electricity or gas in your home?

1. Yes, threatened

2. Yes, shut off

3. No

Have you received fuel assistance in the past 12 months?

1. Yes

2. No

3. Not sure

The next two questions deal with hazards at home.In the past 12 months did you have any of the following problems at home? Please check all that apply. You may click on the checkbox or the words next to it.

1. Roof leaked

2. Problems with electrical wiring

3. No heat for more than 24 hours

4. Water leaks in the home from inside (pipes, sinks, toilets)

5. Water leaks in the home from outside (walls, roof)

6. None of these

In the past 3 months did you have any of the following problems at home? Please check all that apply. You may click on the checkbox or the words next to it.

1. None of the toilets worked

2. Any rats or mice in home or building

3. Any cockroach or insect infestation

4. No running water in the house

5. Broken utilities (sink, dishwasher, etc.)

6. None of these

Do you live in public or subsidized housing, such as a public housing development or an apartment for which you receive housing assistance (Section 8 or housing vouchers)?

1. Yes, public housing

2. Yes, subsidized housing or housing vouchers

3. No

4. Not sure

Thank you for answering our survey.

*END OF SURVEY*

**APPENDIX:**

MSD Scale Questionnaire for other age categories:

If your child is 4-6 months

1. While lying on his/her back and being pulled up to a sitting position, has your child ever held his/her head stiffly so that it DID NOT hang back as he/she was pulled up?

2. Has your child ever laughed out loud without being tickled or touched?

3. Has he/she ever held in one hand a moderate sized object such as a block or rattle?

4. Has your child ever rolled over on his/her own on purpose?

5. Has your child ever seemed to enjoy looking at the mirror at him/herself?

6. Has he/she ever been pulled from a sitting to a standing position and supported (his/her) own weight with legs stretched out?

7. Has your child ever looked around with his/her eyes for a toy which was lost or not nearby?

8. Has your child ever sat alone with no help except for leaning forward on his/her hands or with just a little help from someone else?

9. Has he/she ever sat for 10 minutes without any support at all?

10. Has your child ever pulled himself/herself to a standing position without help from another person?

11. Has your child ever crawled when left lying/on his/her stomach?

12. has he/she ever said any recognizable words such as mama or dada?

13. Has your child ever picked up small objects such as raisins or cookie crumbs only using the thumb and first finger?

14. Has your child ever walked at least 2 steps with one hand held or holding onto something?

15. Has he/she ever waved goodbye without help from another person?

If your child is 7-9 months

1. Has your child ever seemed to enjoy looking in the mirror at him/herself?

2. Has your child ever been pulled form a sitting to standing position and supported his/her own weight with legs stretched out?

3. Has he/she ever looked around with his/her eyes for a toy which was lost or not nearby?

4. Has your child ever sat alone with no help except for leaning forward on his/her hands or with just a little help from someone else?

5. Has your child ever sat for 10 minutes without any support at all?

6. Has your child ever pulled himself/herself to a standing position without help from another person?

7. Has your child ever crawled when left lying on his/her stomach?

8. Has your child ever said any recognizable words such as mama or dada?

9. Has your child ever picked up small objects such as raisins or cookie crumbs using only his/her thumb and first finger?

10. Has your child ever walked at least 2 steps with one hand held or holding onto something?

11. Has your child ever waved goodbye without help from another person?

12. Has your child ever shown by behavior that he/she knows the names of common objects when somebody else names them out loud?

13. Has you child ever shown that he/she wanted something by pointing/pulling or making pleasant sounds rather than crying or whining?

14. Has your child ever stood alone on his/her feet for 10 seconds or more without holding onto anything or another person?

15. Has your child ever walked 2 steps without holding on to anything or another person?

If your child is 10-12 months

1. Has your child ever crawled when left lying on his/her stomach?

2. Has your child ever said any recognizable words such as mama or dada?

3. Has he/she ever picked up small objects such as raisins or cookie crumbs using only his/her thumb and first finger?

4. Has your child ever walked at least 2 steps with one hand held or holding onto something?

5. Has your child ever waved goodbye without help from another person?

6. Has your child ever shown by his/her behavior that he/she knows the names of common objects when somebody else names them out loud?

7. Has your child ever shown that he/she wants something by pulling, pointing, or making pleasant sounds rather than whining?

8. Has your child ever stood alone on his/her feet for 10 seconds or more without holding onto anything or another person?

9. Has your child ever walked 2 steps holding onto anything or another person?

10. Has your child ever crawled up at least 2 stairs or steps?

11. Has your child said 2 recognizable words besides mama/dada?

12. has he/she ever run?

13. has your child ever said the name of a familiar object such as a ball?

14. Has your child ever made a line with a crayon or pencil?

15. Did she/he ever walk up at least 2 stairs with one hand held or holding the railing?

If your child is 13-15 months

1. Has your child every waved good bye without help from another person?

2. Has your child ever shown by his/her behavior that he/she knows the names of common objects when somebody else names them out loud?

3. Has he/she ever shown that h/she wants something by pointing, pulling, or making pleasant sounds rather than crying or whining?

4. Has your child ever stood alone on his/her feet for 10 seconds or more without holding onto anything or another person?

5. Has your child ever walked at least 2 steps without holding onto anything or another person?

6. Has he/she ever crawled up at least 2 steps or stairs?

7. Has your child said 2 recognizable words besides mama or dada?

8. Has your child ever run?

9. Has she/he ever said the name of a familiar object such as a ball?

10. Has your child ever made a line with a pencil or crayon?

11. Did your child ever walk up at least 2 stairs with one hand held or holding the railing?

12. Has he/she ever fed himself/herself with a spoon or fork without spilling much?

13. Has your child ever let someone know without crying that wearing wet (soiled) pants or diapers bothered him/her?

14. Has your child ever spoken a partial sentence of 3 words or more?

15. Has he/she ever waked up stairs by him/her self without holding onto a rail?

If your child is 16-18 months

1. Has your child ever walked up at least 2 steps without holding onto anything or another person?

2. Has your child ever crawled up at least 2 stairs/steps?

3. Has he/she said 2 recognizable words besides “mama/dada”?

4. Has your child ever run?

5. Has your child ever said the name of a familiar object such as a ball?

6. Has she/he ever made a line with a crayon or pencil?

7. Did your child ever walk up at least 2 stairs with one hand held or holding the railing?

8. Has your child ever fed himself with a spoon or fork without spilling too much?

9. Has he/she ever let someone know without crying that wearing wet (soiled) pants or diapers bothered him or her?

10. Has your child ever spoken in a partial sentence of 3 words or more?

11. Has your child ever walked upstairs by himself/herself without holding onto a rail?

12. Has he/she ever walked and dried his/her hands without any help except for turning the water on and off?

13. Has your child ever counted 3 objects correctly?

14. Has your child ever gone to the toilet alone?

15. Has he/she ever walked up stairs by himself/herself with no help, stepping on each step with only one foot?
