# Supplementary material for: The impact of preterm birth <37 weeks on parents and families: a cross-sectional study in the 2 years after discharge from the neonatal intensive care unit
Source: Health Qual Life Outcomes. 2017 Feb 16;15:38. doi: 10.1186/s12955-017-0602-3 (PMC5312577; doi:10.1186/s12955-017-0602-3)
Supplement: Additional file 2: Table S1. — Description of measurement instruments for primary outcomes, modifiable determinants and predisposing characteristics (potential confounders). (DOC 55 kb) [file 12955_2017_602_MOESM2_ESM.doc]

**Additional file 2**: Table S1: Description of measurement instruments for primary outcomes, modifiable determinants and predisposing characteristics (potential confounders)

| Variable | Instrument | Instrument Description |
| --- | --- | --- |
| Primary Outcome Measures |  |  |
| Impact on Family | Impact on Family Scale | 27 item questionnaire with 4 subscales: financial impact, disruption of planning, caretaker burden, and familial burden.  The total negative impact score served as our summary measure of family burden (higher scores indicate greater family burden). |
| Impact on Parent | Infant Toddler Quality of Life Questionnaire Family Burden Scales | Parent impact-emotion scale: The parent impact-emotion domain consists of seven items in which the parent is asked to rate how much anxiety or worry each of the child characteristics described in the items has caused during the past 4 weeks (i.e., feeding/sleeping/eating habits; physical health, emotional well being, learning abilities, ability to interact with others; behavior and temperament).  Parent impact-time scale: The parent impact-time domain consists of seven items in which the parents is asked to rate how much of his/her time was limited for personal needs because of the problems with the child’s personal needs during the past 4 weeks.  For each scale, higher scores indicate less emotional impact and fewer time limitations on the parent. |
| Modifiable Determinants |  |  |
| Use of community based resources | Investigator-created survey | Participants were asked yes/no questions about the use of community-based developmental resources (such as early intervention programs), use of social services such as food assistance programs, Supplemental Nutrition Assistance Program and the Women, Infant, Children’s program as well as energy assistance/disability programs such the Low Income Home Energy Assistance Program, Transitional Aid to Families with Dependent Children, and receipt of Supplemental Security Income (SSI). |
| Financial burden | 2007 Commonwealth Fund Biennial Health Insurance Survey | In addition to questions about employment for the participating parent and his/her partner, we asked 6 yes/no questions from the 2007 Commonwealth Fund Biennial Health Insurance Survey^39-42^ regarding unexpected costs, increased bills, increased out-of-pocket expenses and financial worry. |
| Health related social problems | Helpsteps.com | HelpSteps.com is a survey designed to identify health-related social problems in the following domains: (1) access to health care, (2) housing, (3) food security (4) income security and (5) intimate partner violence |
| Predisposing Characteristics (Potential confounders) |  |  |
| Infant health | Medical record | We obtained information from the medical record regarding delivery and complications during the neonatal hospitalization (“pre-disposing characteristics”). We asked parents questions about their infant’s health status since discharge including the number of emergency department visits, monthly clinic appointments, and hospitalizations, immunizations, dependence on technology, and administration of prescription medications (“post-discharge” characteristics). |
| Infant development | Motor and Social Development Scale | MSD scale was developed by the National Center for Health Statistics to measure motor, social and cognitive development of young children. It comprises 48 items derived from standard measures of child development, including the Bayley Scales of Infant Development, 2nd edition, Gesell Scale, and Denver Developmental Screening Test.^53^ We selected the MSD because it is brief (15 questions) and allows for scoring based on a large, national sample ^52^ with a normative mean of 100 and standard deviation 15, similar to other developmental tests. |
